# Met inhibition revokes IFNγ-induction of PD-1 ligands in MET-amplified tumours

**DOI:** 10.1038/s41416-018-0315-3

**Published:** 2019-02-06

**Authors:** Valentina Martin, Cristina Chiriaco, Chiara Modica, Anna Acquadro, Marco Cortese, Francesco Galimi, Timothy Perera, Loretta Gammaitoni, Massimo Aglietta, Paolo M. Comoglio, Elisa Vigna, Dario Sangiolo

**Affiliations:** 1 Medical Oncology Division, Experimental Cell Therapy, Candiolo Cancer Institute, FPO-IRCCS, Candiolo, Torino Italy; 2 Laboratory of Gene Transfer, Candiolo Cancer Institute, FPO-IRCCS, Candiolo, Torino Italy; 3 Department of Oncology, University of Torino, Candiolo, Torino Italy; 4 Laboratory of Molecular Therapeutics and Exploratory Research, Candiolo Cancer Institute, FPO-IRCCS, Candiolo, Torino Italy; 5 Laboratory of Translational Cancer Medicine, Candiolo Cancer Institute, FPO-IRCCS, Candiolo, Torino Italy; 6 OCTIMET Oncology NV, Turnhoutseweg 30, Beerse, Belgium

**Keywords:** Targeted therapies, Oncogenes, Cancer immunotherapy

## Abstract

**Background:**

Interferon-induced expression of programmed cell death ligands (PD-L1/PD-L2) may sustain tumour immune-evasion. Patients featuring MET amplification, a genetic lesion driving transformation, may benefit from anti-MET treatment. We explored if MET-targeted therapy interferes with Interferon-γ modulation of PD-L1/PD-L2 in MET-amplified tumours.

**Methods:**

PD-L1/PD-L2 expression and signalling pathways downstream of MET or Interferon-γ were analysed in MET-amplified tumour cell lines and in patient-derived tumour organoids, in basal condition, upon Interferon-γ stimulation, and after anti-MET therapy.

**Results:**

PD-L1 and PD-L2 were upregulated in MET-amplified tumour cells upon Interferon-γ treatment. This induction was impaired by JNJ-605, a selective inhibitor of MET kinase activity, and MvDN30, an antibody inducing MET proteolytic cleavage. We found that activation of JAKs/ STAT1, signal transducers downstream of the Interferon-γ receptor, was neutralised by MET inhibitors. Moreover, JAK2 and MET associated in the same signalling complex depending on MET phosphorylation. Results were confirmed in MET-amplified organoids derived from human colorectal tumours, where JNJ-605 treatment revoked Interferon-γ induced PD-L1 expression.

**Conclusions:**

These data show that in MET-amplified cancers, treatment with MET inhibitors counteracts the induction of PD-1 ligands by Interferon-γ. Thus, therapeutic use of anti-MET drugs may provide additional clinical benefit over and above the intended inhibition of the target oncogene.

## Background

Programmed cell death ligand 1 (PD-L1, B7-H1, CD274), programmed cell death ligand 2 (PD-L2, B7-DC, CD273) and programmed cell death receptor 1 (PD-1, CD279) are key modulatory molecules,^1–3^ known as immune-checkpoints, that play a central role at the interface between immune response and tumour microenvironment.^4^ They may significantly impair the ability of the immune system to control tumour progression, therefore therapeutic blocking antibodies have recently been developed.^5,6^ Such checkpoint inhibitors generated impressive clinical results, initially confined to treatment of metastatic melanoma and now progressively extending to other settings like lung, renal, bladder, head and neck and colorectal cancers.^7–12^ The expression of PD-L1 by tumour cells is inducible and interferon gamma (IFNγ) is the most potent inducer, even if other cytokines may have additional effects.^3,13,14^ In a minor fraction of tumours, PD-L1 may also be regulated by intrinsic genetic pathways.^15–17^ T lymphocytes or innate immune cells, which release IFNγ in proximity of the tumours, may give rise to a phenomenon of PD-L1 mediated ‘adaptive resistance’ that dampens the efficacy of antitumour immune response promoting tumour immune-escape. As a consequence, PD-L1 expression is usually clustered in tumour tissues, co-localizing with IFNγ infiltrating lymphocytes.^3,15^ The clinical relevance of PD-L1 expression has been advocated but its clear definition is still object of research and debate. For instance, the presence of PD-L1 in several tumour types including lung, renal, gastric and ovarian cancer has been reported to negatively correlate with patients’ prognosis.^18–22^ Furthermore, a predictive role of PD-L1 expression for clinical response to checkpoint inhibitors has been hypothesised by several studies. It is currently accepted for lung cancer patients^8,23^ but the threshold level or the applicability to other tumour types remains debated.^24,25^ PD-L2 is a second PD-1 ligand, described to be expressed by tumour cells and components of tumour microenvironment.^26^ Similar to PD-L1, PD-L2 is also mainly regulated by interferons and endowed with the ability to inhibit T cell activity and proliferation. Its role in tumour immune-escape is however not as well understood as compared with PD-L1 and currently PD-L2 blocking strategies are not approved in the clinic. Recently, it has become evident that molecular targeted therapies may impact multiple functional interactions between tumour and immune response.^27^ As an example, the treatment with oral inhibitors of the mutated oncogene BRAF, in patients with metastatic melanoma, enhances lymphocyte activation, tumour infiltration and PD-L1 expression,^28^ supporting the exploration of associative clinical trials with checkpoint inhibitors.

The MET oncogene product, the Hepatocyte Growth Factor Receptor (HGFR/MET), emerges as one of the most important oncogenes activated in cancer. MET controls a genetic program, known as ‘invasive growth’, which includes pro-mitogenic, pro-invasive and anti-apoptotic cues.^29^ Genetic lesions within the MET gene results in MET becoming a driver of malignancy. Gene amplification has been documented in cases of patients carrying oesophageal, gastric, and lung tumours, who benefit from anti-MET treatment.^30–32^ Point mutations have been discovered in hereditary and sporadic papillary renal tumours,^33^ and later described in different types of solid cancers, interestingly enriched in frequency in lesions highly metastatic, i.e. Cancer of Unknown Primary origin.^34^ MET exon 14 alterations occur in around the 3% of non-small-cell lung cancer (NSCLC).^35^ These lesions are considered as genomic predictive biomarkers for the use of anti-MET compounds.^36^ Molecular mechanisms involving MET activation have been demonstrated to be drivers of primary/secondary resistance to anti-epidermal growth factor receptor (EGFR), and BRAF-targeted therapies in NSCLC and colorectal (CRC) cancer patient.^37–39^ Also in these cases, the use of MET inhibitors is envisaged.

The current intended aim of anti-MET therapies is to directly negate a driver tumour pathway that sustains its growth, proliferation and dissemination activities. Here we hypothesised that, beside such direct antitumour effects, MET inhibition might interfere with the regulation of PD-1 ligands at tumour level. If confirmed, this would directly endow MET-inhibitors with a potentially beneficial immunologic role which is independent from possible combinations with checkpoint inhibitors. We thus explored the IFNγ-inducible PD-L1/PD-L2 expression in MET-amplified tumours, along with its potential modulation by treatment with MET-inhibitors.

## MATERIALS AND METHODS

### Cell cultures

SNU-5 and Hs746T human gastric carcinoma cells were from ATCC/LGC Standards Srl (Manassas, VA). EBC-1 human lung carcinoma cells and MKN-45 human gastric carcinoma cells were from the Japanese Collection of Research Bioresources (Osaka, Japan). GTL-16 cell line is a clone derived from MKN-45 cells that differs in MET gene copy number from the parental cell line.^40^ All the cell lines are characterised by amplification of the MET gene.^41,42^ Hs746T cells carry also MET Exon 14 skipping.^43^ Cells were cultured as suggested by the supplier.

### Tumour organoids

Human tumour organoids were derived from liver metastasis of colon cancer patients transplanted and expanded in immunodeficient mice (patient-derived xenografts, PDX). The examined specimens belong to a large collection established at the Candiolo Cancer Institute of molecularly annotated PDX^44^ and have been selected according to their genetic make-up. The patients provided informed consent for research use and the study was conducted according to a protocol approved by the institutional review board (ethics committee). Tumours were mechanically disaggregated and three-dimensional cultures of organoids were established by embedding the cells in Growth Factor Reduced Matrigel (Corning Inc., Corning NY). After solidification, Basal Medium—DMEM/F12 (Sigma Life Science, St Louis, MO), plus 2 mM L-glutamine, penicillin–streptomycin (Sigma-Aldrich, St Louis, MO) and N-2 supplement (Life Technologies-GIBCO, Carlsbad, CA)—was added to the cultures. For M162 cultures, Basal Medium was supplemented with 0.4% bovine serum albumin (Sigma-Aldrich), 4 μg/ml heparin (Sigma Life Science), chemically defined lipid concentrate (Life Technologies-GIBCO); for CRC1169 basal medium was supplemented with 2 µM *N*-acetyl-l-cysteine and 20 ng/ml EGF both from Sigma Aldrich.

### Cell treatments

Sub-confluent cell monolayers or organoids were treated with 50 ng/ml of IFNγ-1b (Miltenyi Biotec srl, Bologna, Italy) for 48 h (replaced every 24 h) for protein analysis, or for 3/6 h for mRNA analysis. Cells were also treated with MET specific inhibitors: (a) the ATP-competitive tyrosine kinase inhibitor JNJ-38877605 (JNJ-605) (Selleckchem, Munich, Germany); (b) MvDN30, a chimeric Fab fragment^45^ produced and purified by U-Protein Express BV.^46^ Doses of the inhibitors and treatment combinations are indicated in each specific experiment.

### mRNA analysis

Cellular RNA extracted by using Tri-reagent® (Sigma-Aldrich) was reverse-transcribed into cDNA using the Multiscribe MuLV retrotranscriptase and random primers (ThermoFisher Scientific, Whaltman, MA). cDNA was amplified by Real-time qPCR using Taqman probes and Sso Advanced Universal Probes Supermix (Bio-Rad, Hercules, CA), according to the manufacturer’s protocol. As Taqman probes were used: (a) PD-L1 (CD274, ID Hs00204257-m1); (b) PD-L2 (PDCD1LG2, ID Hs00228839-m1); (c) β-ACTIN (ACTB, ID Hs01060665-g1), all purchased by Thermo Fisher Scientific. PD-L1 and PD-L2 mRNA expression levels were normalised to β-ACTIN expression and reported as fold change between treated and untreated cells.

### Flow cytometry analysis

Cells were detached with Stem Pro Accutase Cell Dissociation Reagent (Thermo Fisher-Scientific) and stained with anti-PD-L1 (PE Mouse anti-Human CD274 IgG1, B7-H1, clone MIH1) or anti-PD-L2 (APC Mouse anti-Human CD273 IgG1, PDCD1LG2, clone MIH18). As isotype controls, cells were stained with PE-anti-Mouse Ig or APC-anti-Mouse Ig antibodies (all the antibodies from BD Biosciences, Franklin Lakes, NJ). Cells were co-stained with DAPI. Expression was analysed by Summit 4.3 software (Dako, Santa Clara, CA). The signal derived from the isotype control was set as: 0 < MFI < 10^1^. Cells were considered PD-L1/PD-L2 positive when MFI > 10^1^.

### Western blotting analysis

Sub-confluent cells were lysed in Laemmli buffer (LB) and 45 µg of total proteins were subjected to SDS-PAGE and western blotting following standard methods. Protein detection was performed by using the following primary antibodies, diluted as indicated: anti-human MET (3D4, 1:3000, Invitrogen Corp., Camarillo, CA), anti-pMET Tyr^1234/1235^ (D26, 1:1000), anti-JAK1 (6G4, 1:1000), anti-pJAK1 Tyr^1022/1023^ (1:1000), anti-JAK2 (D2E12, 1:1000), anti-pJAK2 Tyr^1007/1008^ (1:1000) anti-STAT1 (1:1000), anti-pSTAT1 Tyr^701^ (58D6, 1:1000), anti-PD-L1 (E1L3N, 1:1000), anti-GAPDH (D4C6R, 1:1000) (all from Cell Signaling Technology, Beverly, MA), anti-IFNGR1 (EPR7866, 1:1000, Abcam, Cambridge, UK) and anti-Vinculin (hVIN-1, 1:1000, Sigma Life Sciences). Secondary HRP-conjugated goat anti-mouse IgG (1:20,000) or anti-rabbit IgG (1:20,000) (from Jackson ImmunoResearch, Cambridge, UK) and ECL System (Promega, Madison, WI) were used for protein detection.

### Immunoprecipitation assay

Total cell proteins were obtained by lysis with cold RIPA buffer^47^ in the presence of 1 mM Na_3_VO_4_ and a cocktail of protease inhibitors (all from Sigma-Aldrich). 250 µg of total protein lysates were incubated at 4 °C for 2 h on rotor with anti-human MET DO-24 mAb^48^ covalently conjugated to Sepharose-protein A (GE Healthcare, Buckinghamshire, UK) with Dimethylpimelimidate (Thermo Fisher-Scientific) following standard methods. As control, an equal amount of total proteins was incubated with Sepharose protein A. After five washes with RIPA buffer, immunoprecipitated proteins were eluted with boiling LB and analysed by western blotting.

### Immunofluorescence assay

Organoids were mechanically dissociated and plated on Chamber Slide (ThermoFisherScientific). Three days after plating, organoids were treated as described. Cells were stained with: (a) anti-PD-L1 mAb (E1L3N, 1:200, Cell Signaling) revealed by anti-Rabbit Alexa Fluor 488 secondary IgG Ab (1:400 Thermo Scientific); (b) Alexa Fluor 555-conjugated phalloidin (1:50); (c) anti-pMET Tyr^1234/1235^ mAb (D26, 1:700, Cell Signaling) revealed by anti-Rabbit Alexa Fluor 488 secondary IgG Ab (1:400, Thermo Scientific); (d) anti-human MET mAb (DO-24, 1:50)^48^ revealed by anti-mouse Alexa Fluor 555 secondary Ab (1:400, Thermo Scientific). Cells were counterstained with DAPI. Images were captured with a Leica TCS SP5 AOBS confocal laser-scanning microscope (Leica Microsystems, Wetzlar, Germany). Immunofluorescence acquisition settings were kept constant within each model. MFI was measured by ImageJ software in a fixed area for each channel, background subtracted. PD-L1 MFI was normalised on Phalloidin MFI; phopho-MET MFI was normalised on total MET MFI.

### Statistical analysis

All graphs and statistical analysis were generated using GraphPad Prism 5 software (GraphPad Software, San Diego, CA). MFI values of flow-cytometry experiments were obtained from at least three independent experiments, performed in triplicate. Error Bars represent ± standard deviation (SD). MFI values of immunofluorescence analysis derived from the mean of five different pictures obtained at least from two separated experiments. Pictures are representative images from one experiment. Error bars represent ± standard error of the mean (SEM). Statistical significance was determined using a two-tailed Student’s *t* test (flow-cytometry) and/or Mann–Whitney (immunofluorescence) (*P ≤ 0.05, **P ≤ 0.005, ***P ≤ 0.001).

## RESULTS

### IFNγ upregulates the expression of PD-1 ligands in MET-amplified tumours

**Fig. 1:**
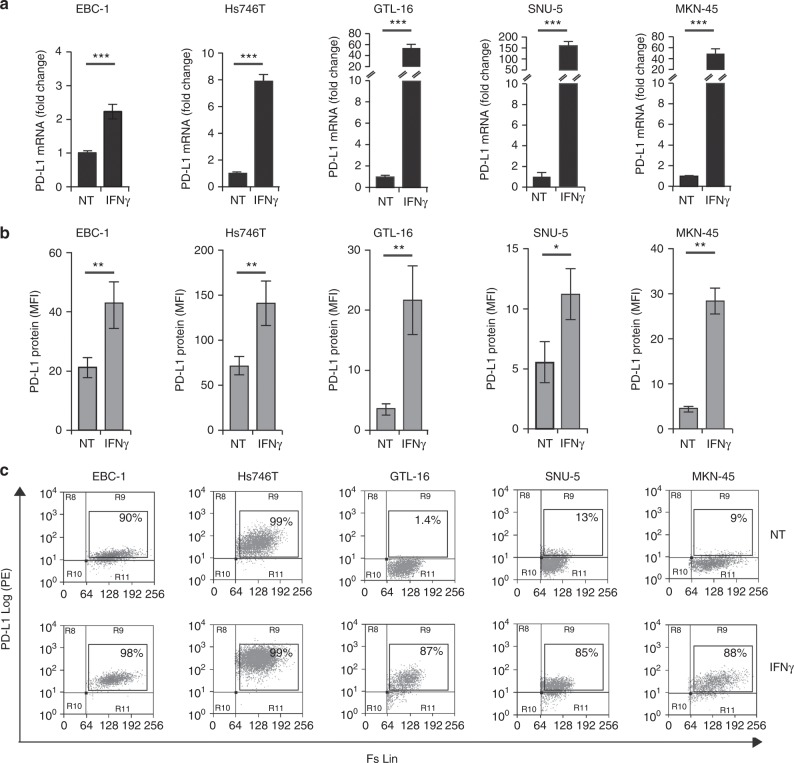
IFNγ treatment upregulates PD-L1 mRNA and protein expression in MET-amplified tumours. **a** Real-time qPCR analysis of PD-L1 mRNA on MET-amplified human cancer cells upon 6 h treatment with IFNγ. Fold change values with respect to untreated controls (NT) reported in the graphs are mean ± standard deviation (SD) calculated from three independent experiments (***, *P* ≤ 0.001). **b** Flow-cytometry analysis of PD-L1 expression on cell membrane of MET-amplified tumours upon 48 h treatment with IFNγ. Mean fluorescence intensity (MFI) values in the graphs are Mean ± SD calculated from three independent experiments (**, *P* ≤ 0.005; *, *P* ≤ 0.05). **c** Representative dot plots from one independent experiment showing the % of viable PD-L1-positive cells in the absence (NT) or presence of IFNγ

**Fig. 2:**
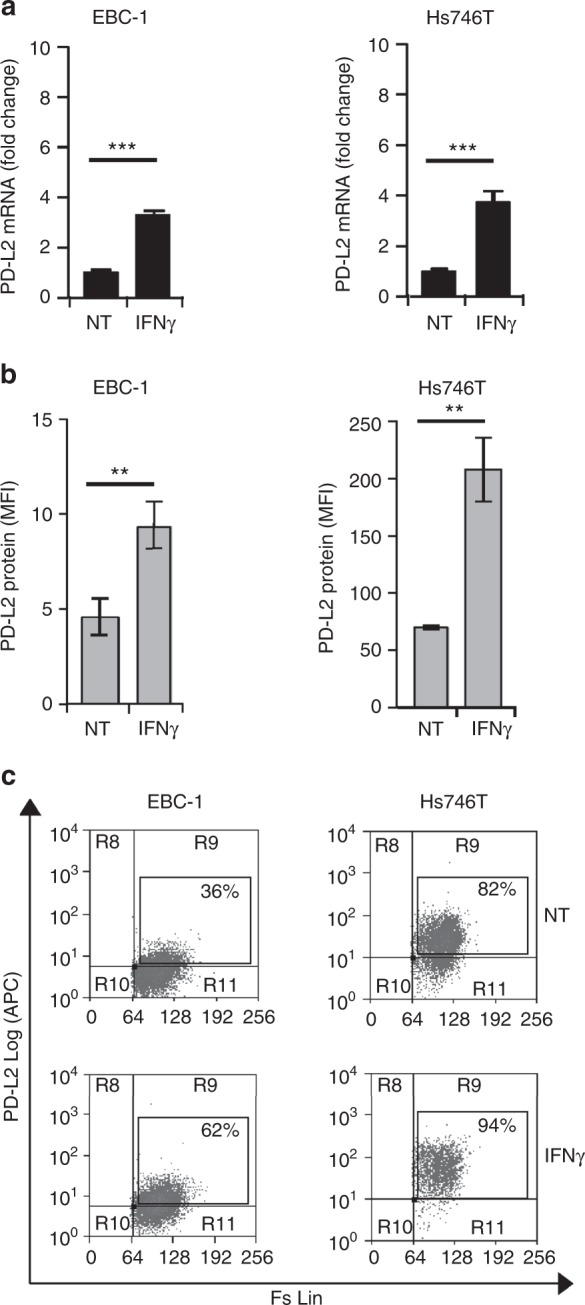
IFNγ treatment upregulates PD-L2 mRNA and protein expression in MET-amplified tumours. **a** Real-time qPCR analysis of PD-L2 mRNA on MET-amplified human cancer cells upon 6 h treatment with IFNγ. Fold change values with respect to untreated controls (NT) reported in the graphs are mean ± SD calculated from three independent experiments (***, *P* ≤ 0.001). **b** Flow-cytometry analysis of PD-L2 expression on cell membrane of MET-amplified tumours upon 48 h treatment with IFNγ. Mean fluorescence intensity (MFI) values in the graphs are Mean ± SD calculated from three independent experiments (**, *P* ≤ 0.005). **c** Representative dot plots from one independent experiment showing the % of viable PD-L2-positive cells in the absence (NT) or presence of IFNγ

A panel of MET-amplified tumour cell lines from different tissue origins has been analysed for IFNγ-inducible PD-L1/PD-L2 expression. PD-L1, variably expressed in unstimulated condition, was consistently upregulated upon exposure to IFNγ. Regulation occurs at the transcriptional level: after 6 h of treatment PD-L1 mRNA increased between 2 and 150 folds, depending on the cell line analysed (Fig. 1a). As a consequence, the membrane expression of PD-L1, determined by flow cytometry on viable cells upon 48 h of exposure to IFNγ, was significantly higher compared with basal levels. In the presence of IFNγ, MET-amplified tumour cells were more than 85% PD-L1 positive, with an increment in mean of fluorescence intensity (MFI) between 2 and 6 folds, depending on the cell line analysed (Fig. 1b, c). The upregulation was dependent on the presence of IFNγ, as we observed that PD-L1 trended to return to basal levels upon 48–72 h from withdrawal of the cytokine (data not shown). An IFNγ-dependent modulation was evident also for PD-L2, in two out four tumour cell lines assessed. In EBC-1 and Hs746T, upon IFNγ treatment, PD-L2 mRNA expression triplicated (Fig. 2a) and protein levels on the cell surface were significantly higher than the basal, as measured by MFI and number of positive cells detected by flow-cytometry (Fig. 2b, c). Tumour cell lines SNU-5 and GTL-16 were not expressing PD-L2, neither under basal conditions nor upon IFNγ stimulation (data not shown).

### Inhibition of MET selectively impairs IFNγ-induced PD-L1/PD-L2 upregulation in MET-amplified tumours

**Fig. 3:**
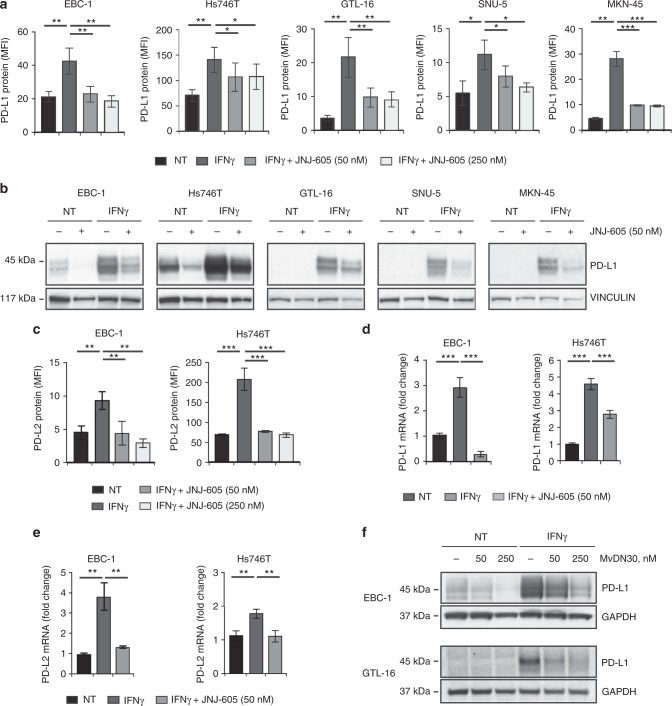
MET inhibitors hamper IFNγ-induced PD-L1 and PD-L2 expression in MET-amplified tumours. **a** Flow-cytometry analysis of PD-L1 protein expression on cell membrane of MET-amplified tumours upon 48 h treatment with IFNγ alone or in combination with the MET-TKi JNJ-605. Mean fluorescence intensity (MFI) values in the graphs are Mean ± SD calculated from three independent experiments (***, *P* ≤ 0.001; **, *P* ≤ 0.005; *, *P* ≤ 0.05). **b** Western blot analysis of PD-L1 expression on total protein extracts obtained from MET-amplified cells treated for 48 h with IFNγ alone or in combination with JNJ-605. As loading control filters were probed with anti-vinculin antibodies (lower panels). Images reported in the figure are representative of results obtained in at least three independent experiments. **c** Flow cytometry analysis of PD-L2 protein expression on cell membrane of MET- amplified tumours upon 48 h treatment with IFNγ alone or in combination with JNJ-605. Mean fluorescence intensity (MFI) values in the graphs are Mean ± SD calculated from three independent experiments (***, *P* ≤ 0.001; **, *P* ≤ 0.005; *, *P* ≤ 0.05). **d** Real-time qPCR analysis of PD-L1 mRNA on MET-amplified human cancer cells upon 3 h treatment with IFNγ alone or in combination with JNJ-605. Fold change values with respect to untreated controls (NT) reported in the graphs are Mean ± SD calculated from three independent experiments (***, *P* ≤ 0.001). **e** Real-time qPCR analysis of PD-L2 mRNA on MET-amplified human cancer cells upon 3 h treatment with IFNγ alone or in combination with JNJ-605. Fold change values with respect to untreated controls (NT) reported in the graphs are Mean ± SD calculated from three independent experiments (**, *P* ≤ 0.005). **f** Western blot analysis of PD-L1 expression on total protein extracts obtained from MET-amplified cells treated for 48 h with IFNγ alone or in combination with the MET blocking antibody MvDN30. As loading control filters were probed with anti-GAPDH antibodies. Images reported in the figure are representative of results obtained in at least three independent experiments

We analysed if the pharmacologic inhibition of MET, currently explored in the clinic as therapeutic option for MET-amplified tumours, could modulate the IFNγ-pathway and consequently PD-L1/PD-L2 regulation. Fourty eight hours treatment in vitro with therapeutic doses of JNJ-605, a small-molecule tyrosine kinase inhibitor (TKi) highly selective for MET,^49,50^ significantly impaired the upregulation of PD-L1 at the cell membrane induced by IFNγ, in all MET-amplified cancer cell lines included in our panel. PD-L1 inhibition, observed by flow-cytometry through the measurement of MFI values (Fig. 3a), was confirmed by immunoblotting analysis (Fig. 3b). Notably, JNJ-605 treatment effectively diminished PD-L1 expression even in the absence of IFNγ treatment, in those tumour cells where a basal amount of protein was present (see EBC-1 and Hs746T, Fig. 3b and Suppl. Figure 1). JNJ-605 modulation occurred selectively in MET-amplified cells, as the induction of PD-L1/PD-L2 by IFNγ was not impaired in tumour cells carrying normal expression of MET, either if the receptor was inactive or if it was activated by chronic exposure to HGF (Suppl. Figure 2). JNJ-605 also strongly counteracted the induction of PD-L2 by IFNγ: upon 48 h of treatment, the ligand exposed at the surface of EBC-1 and Hs746T cancer cells returned to basal levels (Fig. 3c). JNJ-605 action rested on the transcriptional level: upon treatment, a reduction of PD-L1/PD-L2 mRNA molecules produced by IFNγ stimulated cells was observed (Fig. 3d, e). The impairment of IFNγ-induced PD-L1 upregulation was confirmed in selected experiment by treating MET-amplified cells with MvDN30, a MET blocking antibody^45^ (Fig. 3f).

### Inhibition of MET impairs IFNγ pathway interfering with JAK activation

**Fig. 4:**
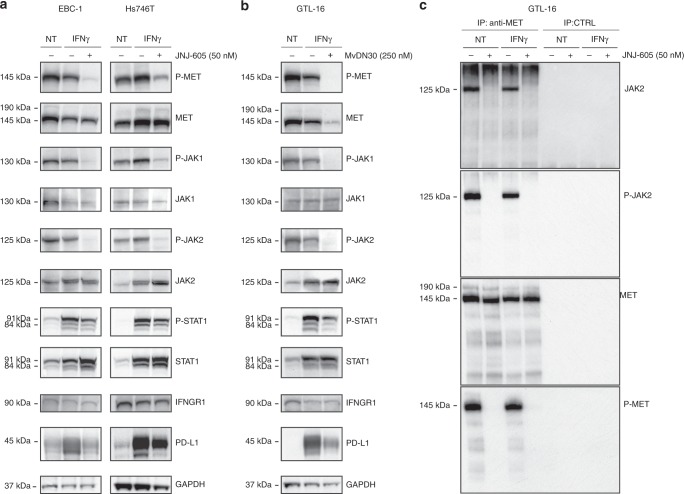
Inhibition of MET impairs IFNγ-induced PD-L1 expression interfering with JAK activation. **a**, **b** Analysis of MET, JAK1, JAK2, STAT1 expression/activation together with IFNGR1 and PD-L1 expression in lysates obtained from MET-amplified cells treated for 48 h with IFNγ alone or in combination with the MET-TKi JNJ-605 (EBC-1 and Hs746T) or with the MET blocking antibody MvDN30 (GTL-16). As loading control filters were probed with anti-GAPDH antibodies. Images reported in the figure are representative of results obtained in at least three independent experiments. **c** Co-precipitation of MET and JAK2 from protein lysates of GTL-16 cells. Total extracted proteins were incubated with anti-MET mAbs cross-linked to Sepharose-ProtA (IP anti-MET) or with Sepharose-ProtA alone (IP CTRL). Proteins eluted from the immunocomplexes were analysed by SDS-PAGE followed by immunodetection with antibodies recognizing JAK2 or phospho-JAK2 (higher panels); as controls, the amount and the phosphorylation of immunoprecipitated MET receptors were also scored (lower panels). Images reported in the figure are representative of results obtained in two independent experiments

In the effort to highlight the possible mechanism underlying the observed modulation of PD-L1 expression, we explored the effect of MET inhibition on the IFNγ-pathway that physiologically drives PD-L1 transcription (Fig. 4a). MET-amplified cancer cells showed highly phosphorylated JAK1/2 kinases, while STAT1 was poorly or not active. Upon stimulation with IFNγ, phospho-JAK1/2 increased or remained unchanged—depending on the cell line—while total STAT1 and phospho-STAT1 were robustly upregulated (Fig. 4a). The pan JAK1/2 inhibitor Oclacitinib-impaired IFNγ induction of PD-L1, confirming the engagement of JAK1/2 in the cellular response to the cytokine (Suppl. Figure 3). When MET inhibitor JNJ-605 was added to cell cultures together with IFNγ, a strong reduction of JAK1/2 activation was observed. This resulted in a significant inhibition of STAT1 activation, concomitantly with an increase of total STAT1 protein. MET inhibition did not impact the total level of cellular receptors for IFNγ (IFNGR1) Fig. 4a). These results, obtained in two distinct MET-amplified cell lines, were confirmed in a third model where MET was inhibited by means of the MvDN30 blocking antibody (Fig. 4b). A direct action of JNJ-605 on JAK2 kinase was ruled out as IFNγ triggered JAK2 phosphorylation was not counteracted by the small molecule in cells expressing inactive MET (Suppl. Figure 4).

To further investigate the relation between MET and JAK, we attempted to see if the two molecules are part of the same signalling complex, by immunoprecipitation of MET and immunodetection with anti JAK2 or phospho-JAK2 antibodies. We observed that the two kinases were associated in dependence of MET phosphorylation (Fig. 4c). This result was confirmed performing the reverse experiment, i.e. immunoprecipitation of JAK2 and immunodetection with anti phospho-MET antibodies (Suppl. Figure 5).

### Inhibition of MET revokes IFNγ-induced PD-L1 upregulation in MET-amplified organoids derived from CRC patients

**Fig. 5:**
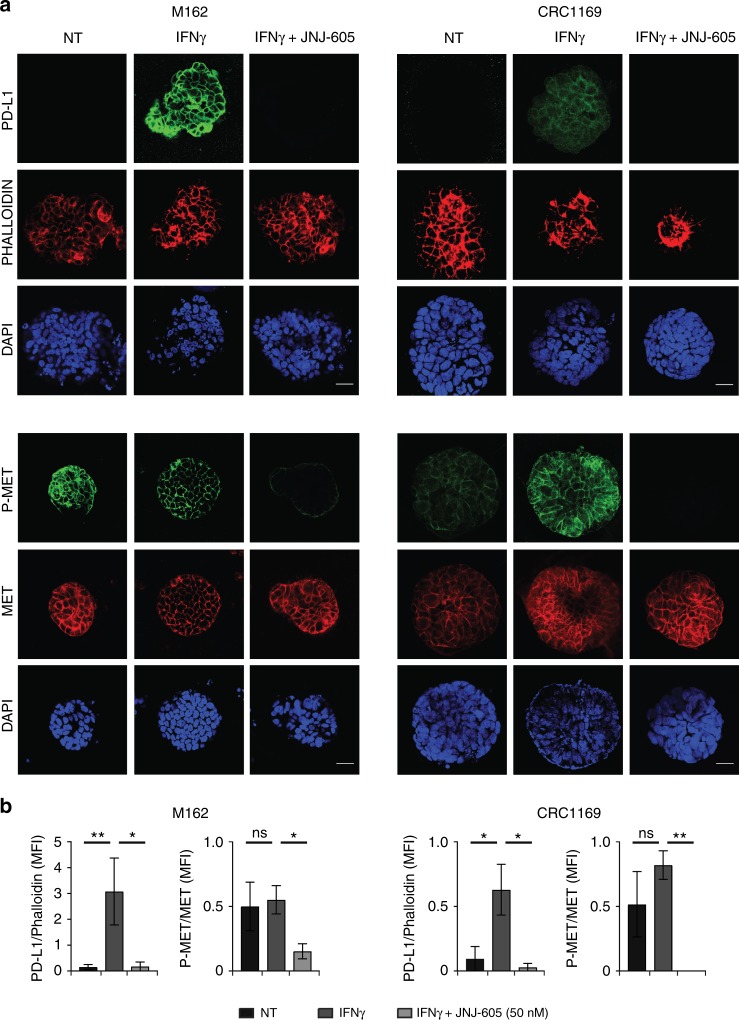
Inhibition of MET impairs IFNγ-induced PD-L1 expression in MET-amplified organoids derived from metastatic CRC patients. **a** Immunofluorescence analysis of PD-L1 expression and phospho-MET status in M162 and CRC1169 organoids treated for 48 h with IFNγ (50 ng/ml) alone or in combination with JNJ-605 (50 nM). NT untreated cells. Upper panels: representative confocal sections showing PD-L1 (green), phalloidin (red) and DAPI (blue). Lower panels: representative confocal sections showing phospho-MET (green), total MET (red) and DAPI (blue). **b** Graphs reporting the ratio of mean pixel fluorescence intensity (MFI) between PD-L1 and phalloidin or the ratio of mean pixel fluorescence intensity between phosphoMET and total MET, background subtracted. Each point is the mean of five values ± SEM. Bar is 50 µm. (**, *P* ≤ 0.01; *, *P* ≤ 0.05; ns not statistically significant)

We then evaluated if MET-inhibition interfered with IFNγ upregulation in human tumour organoids. These three-dimensional cultures of cells embedded into extracellular matrices represent a highly reliable preclinical model, because tumour cells can spontaneously organise, better preserving the original architecture and optimally recapitulating disease-associated changes.^51,52^ We derived human tumour organoids from liver metastasis of colon cancer patients transplanted in immunodeficient mice (patient-derived xenografts, PDX). The examined tumours were selected according to their genetic make-up. M162^37^ and CRC-1169, two different specimens derived from separate patients characterised by MET gene amplification (Suppl. Figure 6), upregulated PD-L1 upon treatment with IFNγ. When exposed for 48 h to JNJ-605, phospho-MET was strongly reduced and PD-L1 expression blunted (Fig. 5a, b). In contrast, M049,^53^ a CRC specimen that does not feature MET amplification (Suppl. Figure 6), responded to IFNγ inducing PD-L1, but JNJ-605 treatment did not result in any PD-L1 downmodulation (Suppl. Fig. 7).

## DISCUSSION

The inducible tumour expression of PD-1 ligands triggered by IFNγ is a significant tumour immune-escape mechanism. In this study we report that the therapeutic treatment with MET inhibitors, in addition to the intended direct antitumour activity against MET-driven cancers, provides a bystander effect revoking the IFNγ-induction of PD-L1/PD-L2 through the inhibition of JAK-STAT pathway.

We confirmed that MET-amplified tumours are susceptible to the inducible expression of PD-1 ligands by IFNγ. Such effect was present in all the analysed models for PD-L1, and in two out of four for PD-L2. Thus, in MET-amplified tumours, adaptive resistance may be in place with possible detrimental effect on antitumour immune response. Therapeutic doses of a small molecule inhibiting MET Kinase activity (MET-TKi) significantly impaired the induction of PD-L1/PD-L2 by IFNγ, apparently interfering with their transcription as supported by the significant reduction in corresponding mRNA levels. We ruled out that the observed modulation was due to a secondary off-target effect of the MET-TKi, as comparable results were obtained also inhibiting the MET pathway by means of a MET blocking antibody that, wiping out MET from the cell surface, acts through a mechanism completely different from the kinase inhibition.^45^ Moreover, the activity of MET inhibitors occurs peculiarly in MET-amplified systems, as IFNγ stimulation of PD-L1/PD-L2 is not revoked by MET-inhibitor treatment in tumour cells that are not MET amplified and express either inactive or HGF-activated MET. This could be due to a different status of the MET kinase that in the case of MET overexpression due to gene amplification results aberrantly strong and persistent, while upon ligand stimulation is tightly regulated and physiologically active.

It is currently not well defined if the therapeutic MET inhibition may or may not favour antitumour immune responses, with preclinical evidence not yet conclusive and sometimes apparently contradictory. A study by Finisguerra et al. suggested that neutrophils with antitumour capacity were dependent on MET/HGF pathway, with their consequent detrimental depletion by treatments with MET inhibitors.^54^ The proposed model however, based on mice reconstituted with upfront MET-deficient hematopoietic cells, did not allow the exploration of myeloid elements within the tumour microenvironment with potentially immune-suppressive functions. In support of this hypothesis, a very recent study reported that MET inhibition hinders tumour migration of immune-suppressive neutrophils, with consequent clinical benefit, enhanced lymphocyte infiltration and potential synergism with checkpoint inhibitors.^55^

Our data fit within this evolving scientific frame, adding the idea that therapeutic MET inhibition may provide, in MET-amplified tumours, an unexpected modulatory activity functionally similar to checkpoint inhibitor antibodies. Furthermore, we showed that checkpoint downmodulation by MET inhibitors may have the advantage of involving both PD-L1 and PD-L2, providing in theory more complete blockage of PD-1 pathways.

Compared to PD-L1, the clinical relevance of PD-L2 is still to be fully grasped and, as now, no clinical data are available about PD-L2 blockade in cancer patients. Even considering the existence of a partial PD-1-independent immune-stimulatory activity operated by PD-L2,^56,57^ its presence at the surface of tumour cells may potentially counteract current therapeutic strategies based on PD-L1 blocking antibodies. We found that only two out four tumour cell lines increased PD-L2 expression upon exposure to IFNγ. This could be due to the fact that PD-L2, differently from PD-L1, may be regulated not only by IFNγ but also by IFNβ, through different transcription factors (IRF-1 and STAT3) binding directly to the PD-L2 promoter.^14^ The efficient activity of MET-inhibitors was however verified in all the cases where IFNγ induced PD-L2 expression.

In clinical perspective, it could be imagined that cancer patients treated with MET inhibitors may benefit from a contextual lowering of the adaptive resistance level mediated by PD-L1/L2. It could result into a facilitation of spontaneous adaptive immune responses, but also be a favourable platform to explore possible synergisms with other immunotherapy strategies. Exploring the immunological relevance of the described effect by MET inhibitors, with the speculated impact on the adaptive immune response, will require further dedicated studies preferably within immunocompetent models. Alternatively, and more relevantly, clinical trials with MET inhibitors will allow to include translational analysis in order to evaluate the rate of PD-L1/L2 expression at tumour sites, along with the corresponding status of lymphocyte activation and infiltration.

Mechanistically, we highlighted that MET inhibition results in the deactivation of JAK1/2, first key molecules downstream of IFNγ receptor, contrasting the activation of STAT1 necessary to trigger the process of PD-L1 transcription. We described for the first time that phosphorylated-MET associates with phospho-JAK2, indicating that they belong to the same signalling complex. This observation is in line with the data reported by Van Schaeybroeck et al. concerning MET influence on the JAK–STAT-3 pathway.^58^ Beside the implications on PD-L1/L2 transcription, our data add a new and potentially relevant piece of information on the complex molecular network of MET, considering the multiple biologic functions mediated by JAK kinases in normal and cancer cells. Conversely to what is observed in cells without a constitutively active MET (see Suppl. Figure 4), MET-amplified tumours show basal phosphorylation of JAKs in the absence of IFNγ. Nevertheless, the activation of these kinases does not translate into a switch-on of the entire pathway, as STAT1 is poorly or not phosphorylated. Only upon IFNγ stimulation STAT1 is strongly activated. JNJ-605 treatment counterbalanced this activation diminishing the level of phosphorylated STAT1, even if total STAT1 proteins increased. This increase is the result of a transcriptional upregulation (data not shown), indicating that rescue circuits are triggered to maintain the IFNγ pathway in an active state. The direct mechanism by which MET inhibition leads to the dephosphorylation of JAK1/2 is still to be determined. On one hand, MET kinase could directly phosphorylate JAKs, contributing to the full activation obtained upon IFNγ stimulation. A second possibility is the activation of a phosphatase consequent to MET inactivation. PTP-1B is a cytoplasmic tyrosine phosphatase known to contribute to the regulation of the JAK/STAT1 pathway by modulating the status of JAK2 phosphorylation.^59^ It has been shown that PTP-1B is associated with MET.^60,61^ PTP-1B catalytic activity is controlled by the phosphorylation of critical residues: when phosphorylated at residue Ser-50 by AKT, the phosphatase activity is down modulated.^62^ PTP-1B is negatively controlled by MET via AKT and might be unleashed upon MET-inhibition, finally determining JAK2 deactivation. Both the mechanisms, either a direct phosphorylation of JAKs by MET or the activation of the PTP-1B—or a different, or more than one phosphatase—could coexist.

In summary, our findings unveil a new, potentially favourable, ‘collateral effect’ that could be associated to the molecular targeted treatment with MET inhibitors in patients with MET-amplified tumours. Revoking one of the main axis that supports the adaptive resistance to antitumour immune responses, anti-MET drugs may provide clinical benefits beyond the intended inhibition of the driver oncogene. Our data provide biological/molecular rational to explore the immune-modulatory effects of MET-inhibition, or possible synergism with adoptive immunotherapy strategies, in clinical trials against MET-amplified tumours.

### Availability of data and materials

Materials and data are available upon request to the corresponding author.

## Electronic supplementary material

supplementary data
