# Supplementary material for: Met inhibition revokes IFNγ-induction of PD-1 ligands in MET-amplified tumours
Source: Br J Cancer. 2019 Feb 6;120(5):527–36. doi: 10.1038/s41416-018-0315-3 (PMC6461865; doi:10.1038/s41416-018-0315-3)
Supplement: Supplementary file 1 — supplementary data [file 41416_2018_315_MOESM1_ESM.pdf]

Supplementary Figure 1

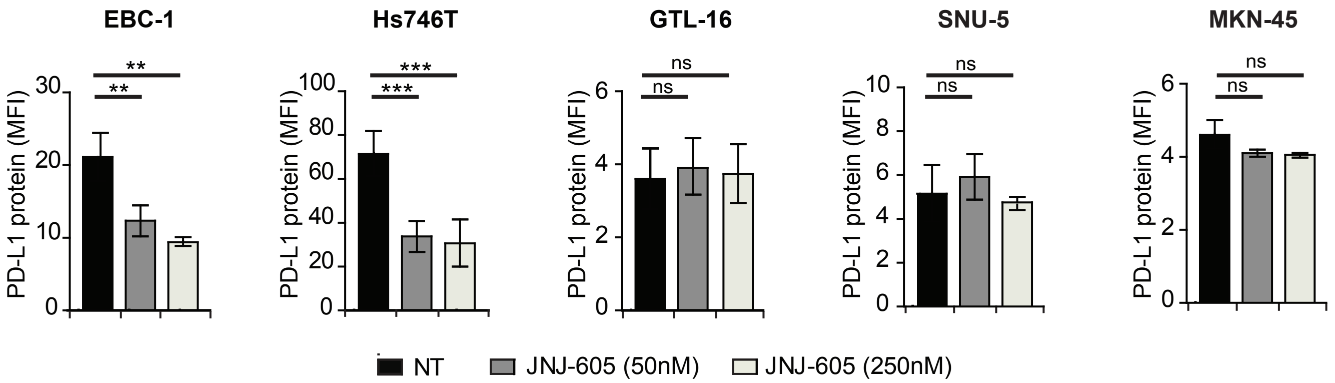

Supplementary Figure 1. MET inhibition reduces PD-L1 protein level in MET amplified tumors carrying basal PD-L1 expression. Flow cytometry analysis of PD-L1 expression on cell membrane of MET-amplified tumors upon 48 hrs treatment with the MET-TKi JNJ-605. Mean Fluorescence Intensity (MFI) values reported in the graphs are Mean ± SD calculated from three independent experiments, performed in triplicate, (**, $P \leq 0.005$; ***, $P \leq 0.001$; ns, not statistically significant).

Supplementary Figure 2

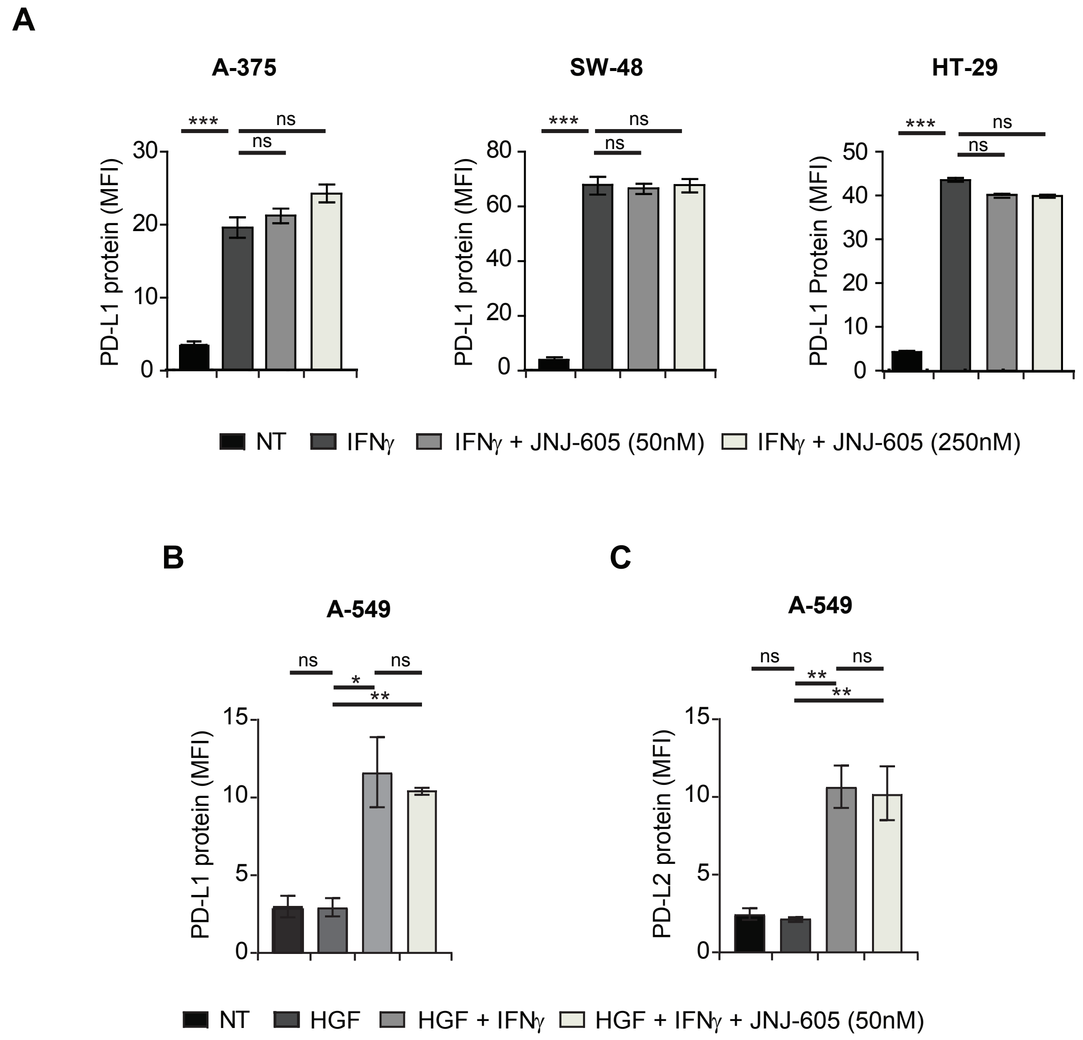

Supplementary Figure 2. Treatment with JNJ-605 does not impair PD-L1/PD-L2 expression induced by IFNγ in tumors cells carrying normal expression of inactive or HGF-activated MET. (A) Flow cytometry analysis of PD-L1 expression on cell membrane of A-375 (human melanoma), SW-48 and HT-29 (human colorectal carcinomas) cells treated for 48hrs with IFNγ alone or in combination with MET-TKi JNJ-605. Mean Fluorescence Intensity (MFI) values reported in the graphs are Mean ± SD calculated from three independent experiments, performed in triplicate. **(B-C)** Flow cytometry analysis of PD-L1/PD-L2 expression on cell membrane of A-549 (human lung carcinoma) cells treated for 48hrs with HGF (50 ng/ml), in combination with IFNγ alone or IFNγ + MET-TKi JNJ-605. Mean Fluorescence Intensity (MFI) values reported in the graphs are Mean ± SD calculated from two independent experiments, performed in duplicate. (***, $P \leq 0.001$; **, $P \leq 0.005$; *, $P \leq 0.05$; ns, not significant).

Supplementary Figure 3

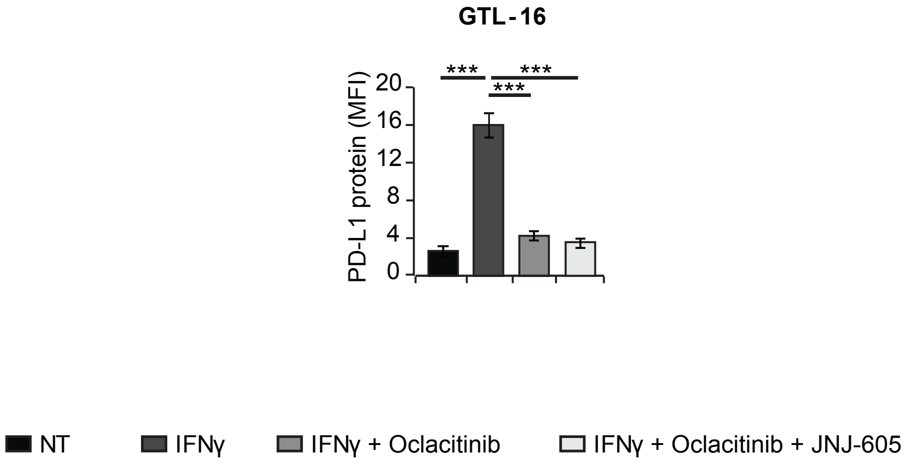

Supplementary Figure 3. Treatment with JAK inhibitor impairs PD-L1 expression induced by IFNγ in MET amplified tumors.

Flow cytometry analysis of PD-L1 expression on cell membrane of MET-amplified GTL-16 cells stimulated with IFNγ and treated with the JAK inhibitor alone (Oclacitinib, 5μM) or in combination with the MET TKi JNJ-605 (250nM). Mean Fluorescence Intensity (MFI) values reported in the graphs are Mean ± SD calculated from three independent experiments, performed in triplicate, (***, $P \leq 0.001$).

Supplementary Figure 4

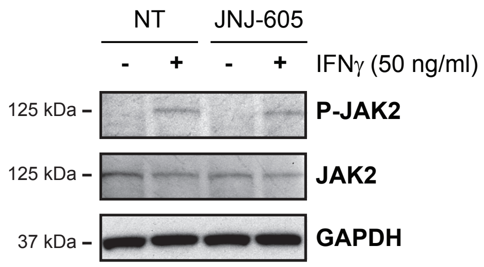

Supplementary Figure 4. MET inhibitor does not impair JAK2 activation in cell expressing inactive MET. Western Blot analysis of JAK2 phosphorylation and expression on total protein extracts obtained from A549 human lung carcinoma cells, treated for 24 hrs with the MET-TKi JNJ-605 (50 nM) and then stimulated for 15 min at 37 °C with IFNγ. As loading control filter was probed with anti-GAPDH antibodies (lower panel). Images reported in the figure are representative of two independent experiments.

Supplementary Figure 5

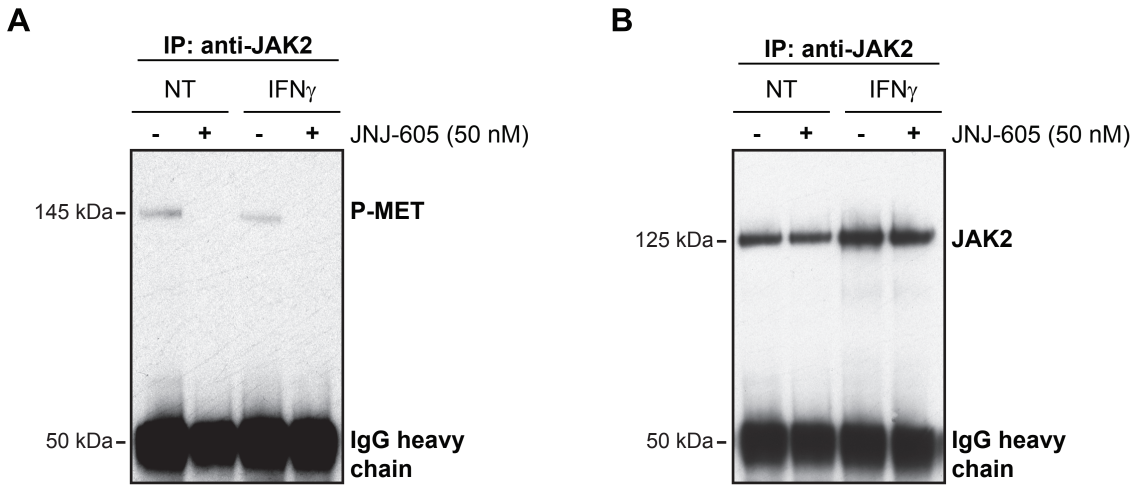

Supplementary Figure 5. JAK2 and MET are associated in the same signaling complex in dependence of MET phosphorylation. Co-precipitation of JAK2 and MET from protein lysates of GTL-16 cells. Total extracted proteins were incubated with anti-JAK2 mAbs conjugated with Sepharose (IP: anti-JAK2). Proteins eluted from the immunocomplexes were analyzed by SDS-PAGE followed by immunodetection with: **(A)** antibodies recognizing phospho-MET protein (P-MET) or **(B)** antibodies recognizing JAK2 proteins. Images reported in the figure are representative of two independent experiments.

Supplementary Figure 6

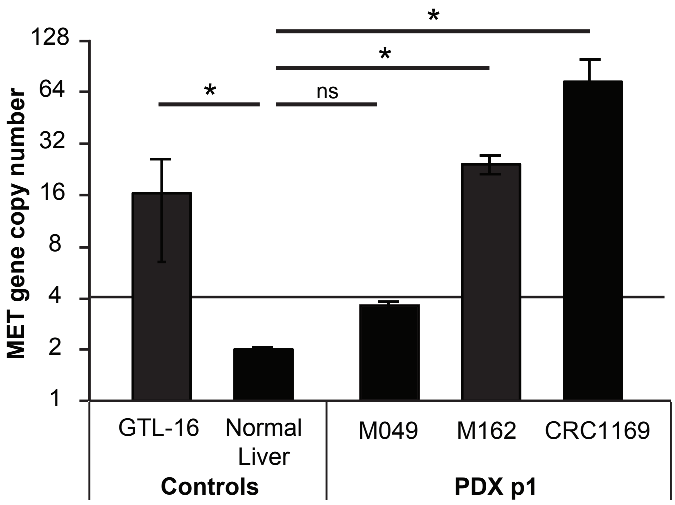

Supplementary Figure 6. MET gene is amplified in M162 and CRC1169 tumors.
MET gene copy number analyzed by Real time qPCR in genomic DNA extracted from human normal liver (as negative control) and from liver metastasis of colon cancer patients at first passage in NOD-SCID mice (patient-derived xenograft, PDX p1). As positive control, MET gene analysis of GTL-16 cells was included. Bars in the graph show the relative MET gene copy number ± SEM. Data were normalized to Normal Liver and to ULK2 (Chr 17) and RPS6KC1 (Chr 1) control genes. The threshold for MET gene amplification was considered 4 gene copies (*, $P \leq 0.05$; ns: not statistically significant).

Supplementary Figure 7

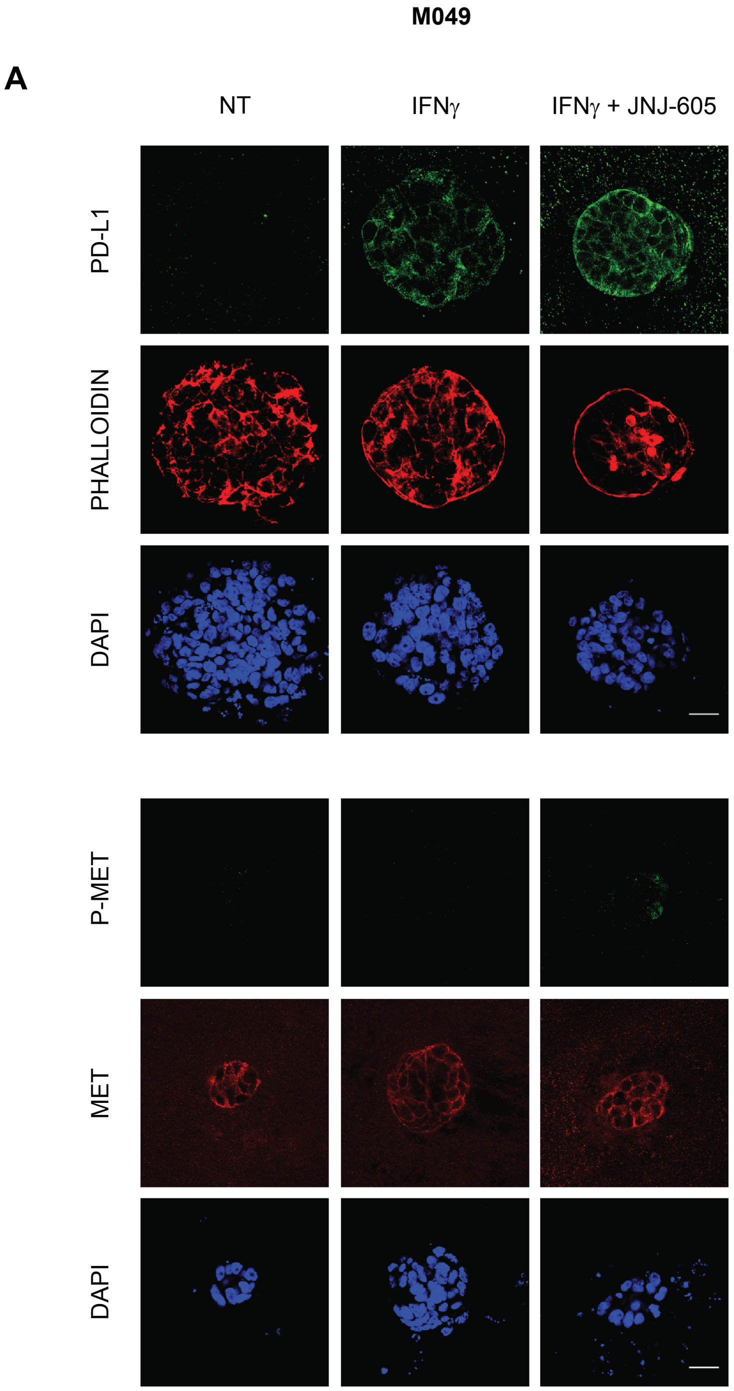

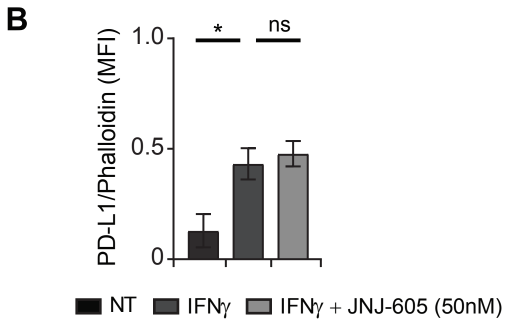

Supplementary Figure 7. Treatment with JNJ-605 does not impair PD-L1 expression induced by IFNγ in organoids derived from a CRC patient carrying normal expression of inactive MET. (A) Immunofluorescence analysis of PD-L1 expression and phospho-MET status in MO49 organoids treated for 48 hrs with IFNγ (50 ng/ml) alone or in combination with JNJ-605 (50 nM). NT: untreated cells. Upper panels: representative confocal sections showing PD-L1 (green), phalloidin (red) and DAPI (blue). Lower panels: representative confocal sections showing phospho-MET (green), total MET (red) and DAPI (blue). Bar is 50 μm. **(B)** Graph reporting the ratio of Mean pixel Fluorescence Intensity (MFI) between PD-L1 and phalloidin, background subtracted. Each point is the mean of 5 values ± SEM. (* $P \leq 0.05$; ns: not statistically significant).

Supplementary Materials and Methods

Cell cultures
A549 (human lung carcinoma), HT29 and SW48 (human colorectal adenocarcinomas) were from ATCC/LGC Standards Srl. All these cell lines featuring diploid MET gene. Cells were cultured as suggested by the supplier.

Tumor organoids
M049 colon cancer spheres were obtained by prof. Carla Boccaccio, Candiolo Cancer Institute, Candiolo (TO) Italy; these tumor cells do not show MET gene amplification. Organoids were generated and maintained as described (Luraghi P, Reato G, Cipriano E, Sassi F, Orzan F, Bigatto V *et al.* MET signaling in colon cancer stem-like cells blunts the therapeutic response to EGFR inhibitors. *Cancer Res* 2014; **74**: 1857-1869).

MET gene copy number analysis
DNA from M162, CRC1196 and M049 PDXs after their first passage in mice (p1) was extracted by the QIAamp DNA Micro kit (c#56304, Qiagen) according to manufacturer's instructions. Quantitative PCR assays were performed using the 7900HT Real Time PCR (Applied Biosystems) and primers for human MET gene (Chromosome 7); primers for ULK2 (Chromosome 17) or RPS6KC1 (Chromosome 1) genes were included as reference controls. PCR was performed in a final volume of 10ul, containing 3µl of DNA (0.67 ng/µl), 5µl of Syber green GoTaq Master Mixes (c#M7122, Promega), 0.72µl of 12.5 µM for each forward and reverse primers and 0.56 µl of water. All reactions were performed in triplicates. DNA was also extracted from GTL-16 cells and from human normal liver tissue, positive and negative controls for MET gene amplification. MET gene copies was calculated by the formula $2*2^{-\Delta\Delta Ct}$.

Primers sequences:
h-MET: Fw primer: 5′- CTTGGTGCAGAGGAGCAATG -3′;
Rv primer: 5′- TGTTTCCGCGGTGAAGTTG -3′,

h-ULK2: Fw primer: 5′- TTTGTGTGTGTGACGGAGTCT -3′;
Rv primer: 5′- AGGCTAAGGCAGGAGAATGAG -3′;

h-RPS6KC1: Fw primer: 5′- GCGGCACTGTGTTGATTACTAC -3′;
Rv primer: 5′- GAGTTGTGGGCTCTGCTAATCT -3′.

Immunoprecipitation Assay
Total protein lysates (500 µg) extracted from GTL-16 cells as described in the main text were immunoprecipitated with Sepharose-conjugated anti-JAK2 mAb (D2E12, 20µl c#4089S, Cell signaling). Immunoprecipitated proteins were eluted and analyzed as described in the main text.

HGF treatment
Sub-confluent monolayers of A549 cells were treated for 48 hrs with 50 ng/ml of human HGF (c#294-HGN-025, R&D Systems Inc., Minneapolis, MN) alone or in combination with 50 ng/ml of IFNγ-1b (Miltenyi Biotec srl, Bologna, Italy) alone or together with JNJ-605 (50nM) for 48 hours (IFNγ was replaced every 24 hours).

JAK inhibitor treatment

GTL-16 cells were pretreated for 30min with the inhibitor of JAK family members (Oclacitinib maleate, Selleckchem c#S8195) and then stimulated with IFNγ alone or co-treated with the MET inhibitor JNJ-605 at the indicated concentrations for 48hrs. As control, cells were stimulated with IFNγ alone.
